# The relationship between adverse childhood experiences and impulsive and risky behaviors: the mediating role of positive and negative emotional motivations

**DOI:** 10.5249/jivr.v15i1.1748

**Published:** 2023-01

**Authors:** Hashem Jebraeili, Shabnam Davudizadeh, Roya Rezaee

**Affiliations:** ^*a*^ Health Psychology, Department of Psychology, School of Social and Educational Sciences, Razi University, Kermanshah, Iran.; ^*b*^ School of Social Sciences and Education, Razi University, Kermanshah, Iran.

**Keywords:** Emotional motivation, Impulsivity, Risky behavior, Self-destructive behavior, Childhood trauma

## Abstract

**Background:**

Although the impact of adverse childhood experiences (ACEs) on healthy behaviors of adulthood is largely investigated, the role of these adversities in a wide variety of impulsive and risky behaviors (RBs) as well as the role of mediating variables has been rarely studied. Therefore, the present study aimed to investigate the mediating role of positive-negativeemotional motivations in the relationship between ACEs and RBs.

**Methods:**

In a cross-sectional study, 401 adults of the general population of Kermanshah (201 individuals) and Kurdistan (200 individuals) were selected and they were assessed using the Risky, Impulsive, & Self-destructive behavior Questionnaire (RISQ) and the Childhood Trauma questionnaire (CTQ). Data were analyzed using latent profile analysis (LPA), the correlation tests and structural equation modeling.

**Results:**

The prevalence of ACEs using LPA was estimated 37.7%. There was a significant correlation between all types of child abuse (not child neglect) and RBs. Emotional motivations played a mediating role in the relationship between ACEs and RBs (RMSEA=0.07, SRMR=0.05, CFI=0.92, TFI=0.90). The proposed model could explain about 11% of the variance of emotional motivations and around 70% of the variance of RBs.

**Conclusions:**

Considering the impact of ACEs on emotional motivations and the impact of emotional motivations on RBs, intervention on emotional motivations may help to reduce RBs in people who suffer from ACEs.

## Introduction

Risky and self-destructive behaviors involve making behavioral choices that may expose people to serious risks of being harmed and they reflect a tendency to engage in harmful behaviors regardless of the potential negative consequences.^1^ According to this definition, a wide range of behaviors can be considered as risky or self-destructive. Some people may put themselves at risk by committing aggressive acts, injuring themselves for a purpose other than suicide, or driving recklessly, for example, while others may risk financially by taking part in large bets or risky investments; some people expose their health to risks by consuming illegal substances or overeating, and others may engage in risky sexual behaviors or commit criminal acts.^2^ Regardless of diversity, by enhancing the likelihood of premature death, long-term disability and mental health consequences, these behaviors, in general, impose great costs on the individual and society.^3,4^ Involvement in risky and impulsive behaviors, no matter they are inherently criminal or not, increases the likelihood of being involved in the judicial system.^5^

Severity of the problems that risky and impulsive behavior imposes on the individual and, on a larger scale, on society highlights the significance of assessing such behaviors and identifying the factors affecting them. One of the factors considered in recent years to recognize the initiation and procedure of RBs is the adverse childhood experiences (ACEs)^6-8^ which is defined as potentially traumatic events that can have long-term negative effects on health and well-being.^9^ ACEs were first defined by Felitti et al.^10^ They describe such experiences as exposure to mental, physical, or sexual abuse and disorders in domestic function, including substance abuse by family members, having members with mental illnesses in the family, witnessing violence against the mother or stepmother and committing criminal behaviors at home during the first 18 years of life. Other studies mention emotional and physical neglect, parental separation, loss of family members or friends, prolonged financial distresses, bullying, community violence, and mass violence or war as ACEs along with other cases named above.^11^

Studies report the high prevalence of ACEs among the general population. A study on 214157 people in the United States, for example, found that over half of the respondents (61.55%) had experienced at least one adverse childhood experience, ^12^ or another study with 12288 participants in the same country found that about 53% of people had experienced at least one childhood trauma^13^ In studies conducted in other countries, the prevalence of ACEs is reported as 46.2% -66.2% in the adult population^14-16^ and 75% -85% among the adolescent population.^17,18^ These statistics reveal the high prevalence of ACEs among adolescents and adults in different countries and cultures, which will have serious consequences for the growth and health of the victims.^7,8,19,20^

The relationship between ACEs and some RBs, including substance and alcohol abuse, has been well reflected in various studies in recent years. Leza, Siria,^11^ for example, in a review of 12 studies that have previously been conducted in the United States revealed that the prevalence of ACE was higher in the population using drugs compared to the general population, and that there was a positive relationship between the number of ACEs and the development and severity of substance abuse in adolescents and adults. This relationship was also depicted in different countries, cultures and groups.^13,21-24^ The relationship between ACEs and some other RBs, such as risky sexual behavior,^25,26^ violence and aggression,^27,28^ and alcohol consumption and dependence^19,20^ was also presented in other studies.

Involvement in RBs occurs in a wide range of situations, for instance, when people are in a negative mood or under the influence of immediate temptations.^29^ In this way, the existing theoretical models identified two initial triggers to reduce or alleviate negative emotional states such as anxiety, sadness or extreme anger^30,31^ and to increase positive emotional states such as pleasing or thrilling experiences^3,32^ as the triggers of RBs. Outstanding models of motivation suggest that there are two primary systems of approach motivation (which involves following rewarding consequences) and avoidance motivation (which involves avoiding disturbing consequences) in which risky and impulsive behaviors are associated with alterations in activation of one or both systems.^33^ For example, studies indicate that substance use is positively related to the approach tendencies such as preparation to seek new rewards or tendencies to thrilling or pleasing states, despite negative consequences.^34^

On the other hand, avoidance tendencies such as the need to confront with negative emotions are also recognized as the primary motivation for RBs.^35^

These two emotional triggers are not independent of each other and these two systems, either alone or in interaction with each other, may lead to RBs.^3^ These systems may also be influenced by childhood experiences. In fact, the evidence suggests that ACEs, affecting the self-regulation processes in general and the emotion regulation process in particular, will lead to an inability to healthy and adaptive adjustment of disturbing emotions and impulses.^36^ Self-regulation processes in childhood and consequently the individual's interactions with the environment are formed in growing children in a way that can profoundly affect the subsequent development and health and longevity in them.^37^ Self-regulation are associated with the brain development in the prefrontal cortex, which are strengthened during several developmental stages and are sensitive to the effects of the upbringing environment.^38^ According to the organizational model of development, behavior regulation is influenced by the characteristics of the upbringing environment at levels of home, school and community.^39^ Children who grow up in stable, responsive, and sensitive domestic environments will develop higher levels of self-regulation, while exposure to inadequate and turbulent upbringing environments would erode self-regulation development and may lead to making decisions based on emotions and impulses in adulthood.^38,40^

Examining the adverse experiences of childhood is highly significant not only because of the wide prevalence, but also due to the extensive impact of such experiences on the growth and health of those experiencing them. The importance of this study becomes doubled particularly when it focuses on the relationship between these experiences and RBs, which can have serious consequences for individuals and society. Although evaluation of such relationships has been highly considered in various studies in recent years, there are still some restrictions. First, those studies mainly focus on a few cases of particular RBs, such as substance and alcohol abuse, and not all RBs have been fully and simultaneously taken into consideration. Second, such studies were mostly conducted in developed countries, and the absence of such studies can still be observed in developing countries, including Iran. Third, although there is a consensus that such experiences do not directly affect health-related consequences,^24^ and some studies have attempted to examine mediating variables,^22,24,41,42^ the mechanism through which childhood experiences can influence RBs is not yet completely known. Therefore, the present study was conducted with three hypotheses to address these deficiencies: first, the amount of reporting ACEs in the present sample is higher than in western studies; second, there is a positive correlation between the number and intensity of ACEs and adult RBs; and third, ACEs lead to an increase in RBs by affecting emotional motivations.

## Methods

**The population, the sample and the sampling method**

The present study was cross-sectional and 401 adults (55% male, 45% female, average age = 31.61) participated in it. Sampling was performed in the spring 2021 in Kermanshah (201 individuals) and Sanandaj (200 individuals), western cities in Iran. The convenience sampling method was applied and the questionnaires were only presented to those who were inclined to complete them and there were no coercions or financial incentives to complete the questionnaires.

**Tools**

**The Childhood Trauma Questionnaire-Short Form (CTQ): **this questionnaire is a 28 items screening tool to identify people with childhood abuse and neglect. It measures five types of childhood misbehavior, including sexual abuse, physical abuse, emotional abuse, emotional neglect, and physical neglect on a 5-point scale from 1 (never) to 5 (always). Its validity and reliability were confirmed in various studies.^43,44^

**Risky, Impulsive, and Self-destructive behavior Questionnaire (RISQ): **this self-report questionnaire contains 38 items that measure the general tendency to engage in risky and self-destructive behaviors in 9 areas of illegal/criminal behavior, substance abuse, aggression, self-harm, gambling, risky sexual behavior, excessive alcohol use, impulsive eating, and driving or spending recklessly.^2^ In each item, participants are asked to report the number of times they have committed these behaviors both in the past month and during their lifetime. In order to reduce the positive skewness, participants' responses to each item are first graded based on five classes (0, 1-10, 11-50, 51-100, and over 100 times), and then the scores of the items in each area are added together to achieve the participant's score in that area. In the questionnaire the participants are also asked to indicate, for each behavior, whether they agree with the statements: " I do this behavior to stop feeling upset, distressed, or overwhelmed" and " I do this behavior to feel excitement, to get a thrill, or to feel pleasure" based on a 5-point Likert scale, from zero (strongly disagree) to 4 (strongly agree). The sum of the participant's answers to the first question of different items would result in the total score of negative emotional trigger of RB and the sum of the participant's answers to the second question of different items would result in the total score of positive emotional trigger of RB. Its validity and reliability were confirmed in Sadeh and Baskin-Sommers^2^ study.

**Procedure**

After preparing the questionnaires, they were presented to the participants (in Persian) in the parks and recreation centers of Kermanshah and Kurdistan, along with some explanations provided about the general objective of the research and that the people were not obliged to participate in the research. Considering that the number of parks and recreation centers in the mentioned cities is not high and in order for the selected sample to be representative of all the people of the city, all parks and recreation centers (with the exception of places where it is not possible to fill out the questionnaire due to the lack of places to sit) were considered as sampling locations. In each of these places, questionnaires were given to people who looked healthy, were willing to fill out the questionnaires, and had sufficient literacy to fill out the questionnaires. In this study, it was attempted to use short questionnaires as much as possible in order not to consume much time to answer them and to avoid respondent fatigue. 

**Data Analysis**

The data collected were analyzed using latent profile analysis (LPA), Spearman correlation test and structural equation modeling by SPSS version 26 and Mplus version 7. In the present study, LPA was used to estimate the prevalence of ACEs. LPA is a kind of person-centered research strategy based on the assumption that there might be several subgroups with distinct characteristics in a particular population and samples extracted from it.^45^ LPA is a model-based technique that provides various statistical indicators based on which individuals can be placed into separate categories according to their behaviors or other traits^46^ and by using this method, we can determine the behavior patterns of people in groups. Structural equation modeling with robust maximum likelihood estimation was used in Mplus software to investigate the mediator role. The reason for using this method is that the normality of the distribution of scores is not required. To increase the simplicity, subscale scores were used as indicators of the factors instead of items. In fact, using 28 items as indicators of ACEs, 76 items as indicators of emotional motivation, and 38 items as indicators of RBs could make it difficult to understand the model. When developing the final model, impulsive eating and physical neglect were excluded from the model due to their low association with own factor. The factor loading of impulsive eating on its respective factor was 0.25 (in comparison, it ranged from 0.49 to 0.82 for other indicators of that factor) and the factor loading of physical neglect on its respective factor was 0.35 (in comparison, it ranged from 0.70 to 0.80 for other indicators of that factor). Positive and negative emotional motivation were also used as two indicators of a latent variable due to high correlation (r=0.80). Positive and negative emotional motivations were also used as two indicators of a latent variable due to high correlation (r=0.80). 

## Results

**Fig. 1:**
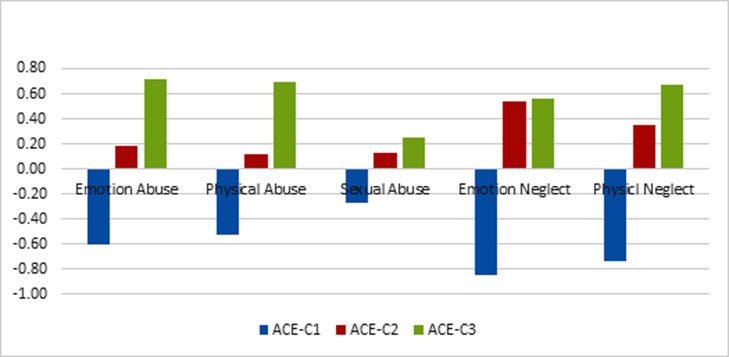
A visual representation of the correlation coefficients between the probability of belonging to each of the groups (classes) and the scores of ACEs.

Demographic data indicated that, out of 401 participants, 221 (55.1%) were male and 178 (44.4%) were female. The average age of participants was 31.61 with a standard deviation of 10.55. Data on ACE (Table 1) revealed that emotional neglect (10.93, 5.43) had been reported as the most prevalent, and sexual abuse (6.01, 2.06) as the least common ACE. Men had experienced more physical abuse (t= 2.28, P=0.023) and physical neglect (t=3.09, P=0.002) compared to women. Latent profile analysis (LPA) was used to assess the prevalence of ACEs with regard to the fact that the questionnaire used did not have a cutoff point. The results of this analysis led to the extraction of three groups (LMR^1^=335.12, P=0.007) (Fig. 1). The first group of people (62.3%) were reported a low rate of ACEs, the second group (28.4%) were reported an average rate of ACEs and these experiences were mainly focused on emotional and physical neglect, and the third group (9.2%) were reported a high rate of ACEs. 

The results of Spearman correlation test (Table 1 ) indicated that there was a significant positive relationship (r=0.11 to r=0.32) between emotional, physical and sexual abuse and all types of RBs. There was a significant relationship between emotional neglect and all types of RBs with the exception of reckless behaviors (r=0.11 to r=0.22). There was also a significant relationship between physical neglect and all types of RBs with the exception of impulsive eating (r=0.11 to r=0.22). There was a significant positive relationship between all types of ACEs, with the exception of emotional neglect, and negative emotional motivation (r=0.16 to r=0.37) and positive emotional motivation (r=0.13 to r=0.27). Among the ACEs, emotional abuse revealed the highest correlation with the total score of RB (r=0.34, P=0.001), followed by physical abuse (r=0.32, P=0.001); in contrast, there was not a significant relationship between emotional neglect and the total score of RB (r=0.08, P=0.104). However, these differences were not statistically significant (Fisher’s z=1.14, P=0.127). Among the RBs, aggression had the highest correlation (r=0.16, to r=0.32) and impulsive eating had the lowest correlation (r=0.01, to r=0.15) with ACEs. Aggression (Fisher’s z=2.05, P=0.020), criminal acts (Fisher’s z=1.69, P=0.046), and self-injury (Fisher’s z=2.02, P=0.022) were more likely to be influenced by ACEs than impulsive eating. Risky sexual behavior had the highest correlation with sexual abuse (r=0.32; Fisher’s z=2.04, P=0.021) in compare to other ACEs (average correlation=0.19). Substance abuse (r=0.51, P=0.001), self-harm (r=0.40, P=0.001), impulsive eating (r=0.61, P=0.001) and criminal acts (r=0.47, P=0.001) were mostly associated with negative emotional motivation, and aggression (r=0.56, P=0.001), gambling (r=0.40, P=0.001), risky sexual behavior (r=0.41, P=0.001), excessive alcohol use (r=0.39, P=0.001) and reckless behavior (r=0.59, P=0.001) were mainly related to positive emotional motivation. Childhood abuses compared to neglect (Fisher’s z=2.92, P=0.002), have a high correlation with emotional motivations.

**Table 1:** correlation coefficients between variables.

Variables	Mean	Standard deviation	E. abuse	P. abuse	S. abuse	E. neglect	P. neglect	NEM	PEM
Emotional abuse	4.47	3.18							
Physical abuse	6.77	3.09	.56**						
Sexual abuse	6.01	2.06	.44**	.43**					
Emotional neglect	10.93	5.43	.47**	.49**	.20**				
Physical neglect	8.57	4.00	.48**	.50**	.21**	.74**			
Negative E. Motivation	15.03	20.54	.37**	.34**	.26**	.06	.16**		
Positive E. Motivation	12.80	18.52	.27**	.28**	.18**	.00	.13*	.80**	
Drug use	0.52	1.48	.25**	.26**	.16**	.14**	.16**	.51**	.47**
Aggression	0.63	1.22	.31**	.32**	.16**	.19**	.22**	.52**	.56**
Gambling	0.30	0.79	.18**	.25**	.18**	.14**	.13*	.30**	.40**
Risky sexual behavior	0.22	0.73	.21**	.22**	.32**	.09	.11**	.36**	.41**
Heavy alcohol use	0.21	0.57	.21**	.20**	.18**	.19**	.17**	.37**	.39**
Self-harm	0.36	0.95	.28**	.28**	.28**	.18**	.17**	.40**	.32**
Impulsive eating	1.68	2.37	.15**	.11*	.11*	-.12*	.01	.61**	.47**
Reckless behavior	1.01	1.21	.17**	.20**	.16**	.01	.11*	.53**	.59**
Crime	0.31	0.82	.25**	.25**	.24**	.17**	.18**	.47**	.44**
Total Risky behavior	5.32	6.56	.34**	.32**	.25**	.08	.16**	.86**	.78**

**P<0.01 *P<0.05

**Figure 2:**
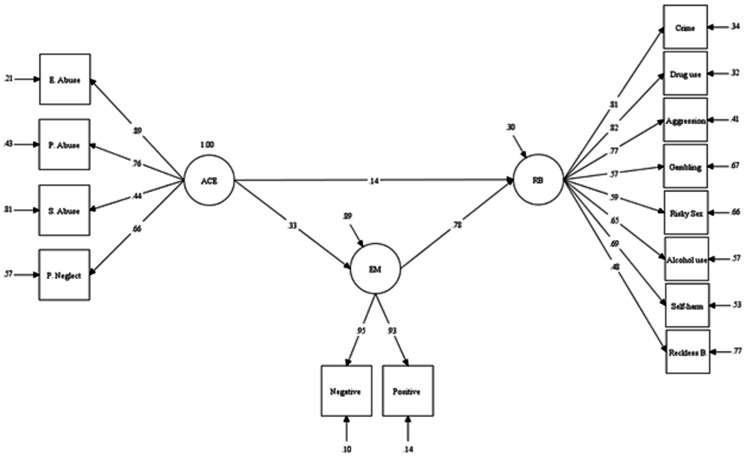
The mediating role of emotional motivations in the relationship between ACEs and RBs.

Results of structural equation modeling using the robust maximum likelihood estimation (with exclusion of impulsive eating and physical neglect) (Fig. 2) confirmed that the model is well fitted with the data (SRMR=0.05, RMSEA=0.07, CFI=0.92, TFI=0.90). ACEs would lead to RBs directly (β=0.14, P=0.049) and/or through increasing emotional motivation (β=0.26, P=0.001). This model could also explain about 11% of the variance of emotional motivations and about 70% of the variance of RBs.

1. adjusted Lo–Mendell–Rubin likelihood ratio test

## Discussion

In the present study, LPA was used to estimate the prevalence of ACEs. Applying this method led to extraction of one group with low ACEs and two groups with medium and high ACEs (three groups in total). Considering that 62.3% of people were categorized in the group with low ACEs and the rest of individuals were placed in the other two groups, it can be concluded that the prevalence of ACEs in the present sample is at least 37.7%. Despite using a different method to estimate the prevalence of ACEs in the present study, this percentage is relatively similar to the value obtained in other studies.^15,16^ In comparison to women, men reported more experiences in the two areas of physical abuse and neglect, which is consistent with previous studies.^21^ This finding can indicate a higher prevalence of this type of ACE in men, or it may indicate that men are more likely than women to reveal such experiences.

The correlation test indicated that there is a relationship between ACEs and most of RBs. But not all ACEs did play the same role in forming these behaviors; so that there was the highest correlation between the physical and emotional abuse and different types of RBs while emotional neglect showed the lowest correlation with these behaviors. Although these differences were not statistically significant, it still seems logical that child abuse, due to the severity of the trauma, has a higher correlation with RBs compared to neglect. So that early studies focused mainly on the role of abuse in developmental disorders in children and its consequences in their life.^10^ The relationship between ACEs and RBs was confirmed, but what is more important is the path and mechanism by which ACEs lead to RBs. The correlation results showed that the ACEs, especially childhood abuses compared to neglect, have a high correlation with emotional motivations and probably lead to RBs through the change of these motivations. This finding is consistent with the results of structural equation modeling in which the mediating role of emotional motivations was confirmed in the relationship between ACEs and RBs suggests that ACEs, particularly child abuse, would lead to appearing disorders in the growing child's tendency system (towards rewards) and avoidance (of punishments), which in turn will show itself as RBs in the adulthood.

In this regard, emerging developmental approaches suggest that children and adolescents that are exposed to tough and unpredictable environments of upbringing would develop cognitive preferences for short-term versus long-term rewards.^47^ In a stressful environment where there is resource and opportunity scarcity and they are unpredictable to appear, there is little or no reinforcement to delay satisfaction in the hope of greater rewards in the future. Over time, the growing child learns to prefer instant rewards and this would result in a tendency to make impulsive decisions.^38^ It is assumed that this tendency for instant reward in people along with ACEs would make them seek excitement or pleasing states constantly, despite the negative consequences, on the one hand,^34^ and, on the other hand, it would cause them to be unable to tolerate negative emotions and to seek the quickest solution to relieve;^35^ both these problems can result in RBs. 

RBs were different from each other in terms of the degree of being influenced by ACEs. For example, aggression, criminal acts, and self-injury are more likely to be influenced by ACEs than impulsive eating. Although the association of ACEs with some RBs such as substance use,^13,21-24^ aggression,^27,28^ risky sexual behavior^25,26^ and alcohol use^19,20^ has been shown in previous studies, the association of ACEs with other behaviors such as gambling, criminal acts, and self-harm has been less investigated. These results indicate a wide range of consequences associated with ACEs. Intervening on these behaviors requires considering and neutralizing these consequences. Furthermore, some of these behaviors were specifically related to certain types of ACEs; compared to other behaviors, risky sexual behavior, for instance, had the highest correlation with having the experience of sexual abuse. This finding shows the specific action mechanism of some ACEs, but how and in what way childhood sexual abuse leads to adult sexual behavior needs further study. Perhaps using interviews to find out the beliefs of people with these experiences can be helpful. 

There are limitations in present study that can restrict its generalizability: first, regarding the fact that participation in the present study was voluntary, the participants may not present real examples of society; second, considering the data collection method, which was self-reporting, samples might not have provided accurate information about their behaviors and experiences; Also, considering that the current research was cross-sectional and correlational, longitudinal research is needed for causal conclusions.

## Conclusion

As expected, the results indicated a high prevalence of ACEs. Although many people, especially women, may not be willing to share their childhood experiences with others, about 38% of people reported varying degrees of ACEs. This finding highlights the need to pay attention to parenting and parent education in the country. It also shows the importance of the government's attention to the family and support for families that are dysfunctional for various reasons, and legal support for the children who live in these families. In addition, providing support and treatment facilities for women who suffer from these experiences can reduce the effects of these experiences on their own parenting style and prevent the intergenerational transmission of defective parenting style. The fact that these experiences are less reported in women than in men can also be a tip that the reported statistics may be much lower than the actual amount and it points out the need to pay attention to the signs of these abuses in women in clinical settings.

The findings also showed that ACEs are related to a wide variety of RBs and lead to these behaviors through changes in emotional motivations. RBs include a wide range of behaviors, most of which are closely related and are influenced by ACEs. But there are exceptions here, for example, impulsive eating had little correlation with other RBs and was therefore excluded from structural equation modeling. This behavior had little connection with ACEs, and the reason for doing this should be looked for somewhere else. In the structural equation modeling, emotional neglect was also removed from the model because its correlation with the total score of RB was insignificant. This shows that all ACEs are not of equal importance in predicting RBs, and all types of abuse are more difficult experiences than emotional neglect. The implications of these results are that, first, professionals who work with children should be sensitive to the signs of possible child abuse. This is especially important in Iran, where professionals may not want to enter the privacy of the family and report possible abuses. This is especially important in our country, where professionals may not want to enter the privacy of the family and do not want to report possible abuses. In addition, the treatment services that are provided for people with RBs should be accompanied by considering and neutralizing the effects of childhood abuse. In this regard, it is recommended for future researches to investigate the relationship between adverse childhood experiences and emotional motivations in more depth and if possible with longitudinal studies. Also, designing interventions to reduce the effects of adverse childhood experiences on emotional motivations can be useful.

**Acknowledgements**

H. J. conceived and designed the evaluation and drafted the manuscript. S. D. &R. R. participated in designing the evaluation, performed parts of the statistical analysis and helped to draft the manuscript. H. J. re-evaluated the clinical data, revised the manuscript and performed the statistical analysis and revised the manuscript. S. D. &R. R. collected the data. H. J. re-analyzed the clinical and statistical data and revised the manuscript. All authors read and approved the final manuscript.
